# An audit on prescribing practice and risk of serotonin syndrome among patients with chronic pain

**DOI:** 10.1192/bjo.2021.425

**Published:** 2021-06-18

**Authors:** Salakan Rai, Aizad Yusof

**Affiliations:** Walsall Healthcare NHS Trust

## Abstract

**Aims:**

To determine the incidence of prescribing practice with associated risk of serotonin toxicity among patients with chronic pain conditions.

**Background:**

Serotonin syndrome is a potentially life-threatening condition caused by excessive serotonergic activity, usually from drug interactions. Concurrent use of antidepressants is strongly linked to serotonin syndrome, with recent data revealing record numbers of NHS prescribed antidepressants in 2019. Antidepressant medications are also used in chronic pain management for their anti-neuropathic pain properties. However, it is well-recognised that a significant number of chronic pain patients suffer from anxiety and depression. This cohort of patients is therefore vulnerable to being exposed to multiple concurrent antidepressant agents, and thus at relatively higher risk of serotonin syndrome compared to other patient groups. Additionally, these patients are likely to be exposed to the concurrent use of antidepressants and certain analgesic agents particularly phenylpiperidine derivatives which increases serotonin toxicity risk.

**Method:**

Medications of patients presenting to a secondary care pain clinic within the last year were looked into. Patients were selected at random by pain management secretaries. Concurrent use of multiple antidepressant agents including Selective Serotonin Reuptake Inhibitors (SSRIs), Serotonin Noradrenaline Reuptake Inhibitors (SNRIs), Tricyclic Antidepressants (TCAs) or Tetracyclic Antidepressant (TeCA) was noted. Additionally, concurrent use of any of these antidepressant agents and phenylpiperidine derivatives such as Fentanyl and Tramadol was noted.

**Result:**

Data on medications of 97 patients were collected. A total of 28 patients (28.8%) were observed to have at-risk medication combinations. Out of these, five patients were on both SSRI and TCA. Two patients were on both TCA and TeCA. Four other patients were on either a combination of SSRI and SNRI, SNRI and TCA, SSRI and TeCA, or TCA and TCA. Three patients were on both Fentanyl patches and an antidepressant. Fourteen patients were on both an antidepressant and Tramadol. None of these patients were diagnosed with serotonin syndrome; however, it is unclear as to whether these patients experienced milder symptoms of the syndrome.

**Conclusion:**

A considerable number of patients in this group were on medication combinations putting them at risk of serotonin syndrome. Despite no documented patient harm, there is an urgent need for an increased awareness among prescribers on drug interactions which may lead to this syndrome and a subsequent change in prescribing practice.

